# Differences in gut microbes in captive pangolins and the effects of captive breeding

**DOI:** 10.3389/fmicb.2022.1053925

**Published:** 2022-12-06

**Authors:** Wenjing Jiao, Lina Liu, Zhiliao Zeng, Linmiao Li, Jinping Chen

**Affiliations:** ^1^Guangdong Key Laboratory of Animal Conservation and Resource Utilization, Institute of Zoology, Guangdong Academy of Sciences, Guangzhou, China; ^2^Shenzhen Management Bureau of Natural Reserve, Guangdong, China

**Keywords:** adaptive environment, captive breeding, conservation biology, gut microbiota, pangolins

## Abstract

Intestinal microorganisms are crucial for health and have a significant impact on biological processes, such as metabolism, immunity, and neural regulation. Although pangolin are protected animals in China and listed as critically endangered (CR) level by The International Union for Conservation of Nature (IUCN), the population of wild pangolins has decreased sharply in recent decades. Captive breeding has been adopted to protect pangolins, but the survival is low due to gastrointestinal infections, diarrhea, and parasitic infections. Studies on intestinal microbes in pangolins may reveal the relationship between intestinal microorganisms and health and assist protection. To explore the relationship between intestinal microorganisms and pangolin health, blood parameters and intestinal microorganisms of 10 pangolins (two *Manis pentadactyla* and eight *Manis javanica*) were studied at the Shenzhen Wildlife Rescue Center. There is difference among adult Sunda pangolins (*M. javanica*), adult Chinese pangolins (*M. pentadactyla*) and sub-adult Sunda pangolins (*M. javanica*) in intestinal microbial composition, diversity and phenotypic diversity, which suggested that adult Sunda pangolins occupied more diversity and proportion of microbial species to resist environmental pressure than the others. Due to the captive breeding serum cortisol of pangolins was increased, and the intestinal microbial structure changed, which may affect immunity. This study provides a scientific basis for the rescue of pangolins through artificial breeding.

## Introduction

The wild population of pangolins has decreased due to illegal hunting and loss of habitat (Heinrich et al., 2016). In February 2021, the pangolin was listed as one of the first-class protected animals in China [Ministry of Agriculture and Rural Areas of the State Forestry and Grassland Administration (Announcement No. 3 of 2021)] (http://www.forestry.gov.cn/). Currently, there is a scarcity of in-depth research on the ecology and biology of the wild Chinese pangolin, and the pangolin's low reproduction rate, diet specificity, and unique habitat requirements make *ex situ* protection of this species challenging (Hua et al., 2015). However, some studies have improved the survival of captive pangolins by solving digestion problems in artificially raised Chinese pangolins (Yang et al., 2007). Nevertheless, there is currently no long-term captive pangolin population. The main reasons for the deaths of artificially bred pangolinsare gastrointestinal infections, diarrhea, and parasitic infections (Khatri-Chhetri et al., 2016; Mohapatra et al., 2016; Jabin et al., 2019; Liu et al., 2019). Additionally, pangolins are prone to stress responses due to hunting as well as breeding environments (Yan et al., 2021). This may cause chronic long-term stress during captivity. When cortisol (the stress hormone) is secreted in large quantities, it may give rise to immune suppression and hence reduce immunity (Fast et al., 2008; McGregor et al., 2016). Studies have also shown that elevated cortisol levels are associated with changes in oral and intestinal microbiota (Jiang et al., 2015; Duran-Pinedo et al., 2018).

Intestinal microorganisms play an important role in the activities of the nervous and immune systems, including the regulation of health, cognition, behavior, and emotions of animals (Abt et al., 2012). Many studies have shown that the response of cortisol to stress is closely related to the intestinal microbiota. Exposure to stress in early life or adulthood has been reported to change the microbiota, thus affecting the stress response (De Palma et al., 2015; Golubeva et al., 2015; Jasarevic et al., 2015; Bharwani et al., 2016). The response of cortisol to stress was positively correlated with the abundance of some bacteria. In peripubertal children, cortisol levels in hair have been linked to variation in *Bacteroidales* S24-7 uncultured, *Sediminibacterium, Lachnospiraceae* UCG005, *Ruminococcaceae* uncultured, *Phascolarctobacterium, Acidaminococcaceae, Alcaligenaceae*, and *Burkholderiales* (Michels et al., 2019). Serum cortisol mediates the connection between fecal *Ruminococcus* and the levels of n-acetylaspartate (NAA) in the brain (Mudd et al., 2017). The immune system plays an important intermediary role in the dynamic balance between the brain and the intestine (Bengmark, 2013). The hypothalamus–pituitary–adrenal axis, autonomic nervous system, and enteric nervous system interact directly with the immune system (Genton and Kudsk, 2003; Leonard, 2006, and Nance and Sanders, 2007). One study indicated that the cortisol level observed in Malayan pangolins rescued from the wildlife trade was significantly lower than those raised in captivity. Moreover, significant differences in the composition and diversity of intestinal microbiota between the two groups were also reported (Yan et al., 2021).

Hormones mediate changes in intestinal microorganisms in response to environmental changes (Mudd et al., 2017; Khakisahneh et al., 2021). Therefore, it can be inferred that captive pangolins under chronic stress may experience an increase in serum cortisol, which changes their intestinal microbiota, resulting in reduced immunity and increased incidence of disorders. This study uses the same batch of captive pangolins as research subjects, and their blood indexes, serum cortisol, and intestinal microorganisms were detected and analyzed to verify the above hypothesis. The purpose of this study was to determine the key factors affecting the health of captive pangolins and the corresponding countermeasures.

## Materials and methods

### Ethical approval

The procedures for the care and use of animals were approved by the Ethics Committee of the Institute of Zoology, Guangdong Academy of Sciences (Guangzhou, China), and all applicable institutional and governmental regulations concerning the ethical use of animals were followed.

### Sample collection

This study was carried out at the Shenzhen Wildlife Rescue Center, located in Shenzhen, Guangdong, China. The pangolins were kept separately, each inhabiting a small enclosure in a 5 m^2^ room. The temperature was maintained at 20–25°C. The diet consisted mainly of black ants (*Polyrhachis vicina*), corn, some vitamins, and water. Eight adult pangolins were raised for ~6 years, and other sub-adult pangolins born in the rescue center in 2019 and 2020 were also raised. Details regarding samples from the 10 pangolins included in the study, which included two adult Chinese pangolins (*Manis pentadactyla*), six adult Malayan pangolins (*Manis pentadactyla*), and two sub-adult Malayan pangolins, as well as grouping, are shown in Supplementary Table 1.

Blood was collected from the coccygeal vein along the ventral midline of the tail, after disinfecting the skin between the scales with 70% ethanol. A volume of 3–5 ml of whole blood was collected using a 10 ml syringe. Blood for hematology (1 ml) and serum biochemistry studies (2–5 ml) was collected in MiniCollect^®^blood collection tubes containing ethylenediaminetetraacetic acid and plain blood collection tubes, respectively. Hematology samples were processed within 24 h of collection, while serum biochemistry samples were sent to the laboratory within 0.5 h of collection and were centrifuged to extract the serum samples, which were then tested.

Rectal swab samples collected from the pangolins were immediately frozen in liquid nitrogen and stored at −80 °C until DNA extraction. Detailed information regarding these samples is shown in Table 1. DNA extraction was performed using the Magpure Stool DNA kit (Magen, Guangdong, China) as per the protocol recommended by the manufacturer.

**Table 1:** Statistics of species annotation.

	**Sample**	**Phylum**	**Class**	**Order**	**Family**	**Genus**	**Species**
Group 1	SZS1	6	10	19	28	42	63
	SZS2	4	7	14	22	33	52
Group 2	SZS3	6	10	16	28	49	72
	SZ03	4	10	22	41	65	113
	SZ11	5	9	20	33	49	75
	SZ15	9	15	29	45	59	76
	SZD1	5	10	19	35	51	74
	SZD3	5	9	22	40	75	130
Group 3	SZSWZ	4	8	20	29	51	77
	SZXTY	11	20	44	69	101	132
	Total	14	24	52	93	173	274

### High-throughput sequencing and bioinformatics analysis

After DNA extraction, specific primers (27F: AGRGTTTGATYNTGGCTCAG and 1492R: TASGGHTACCTTGTTASGACTT) with barcodes were synthesized according to the full-length primer sequence. Polymerase chain reaction amplification was carried out, and the products were purified, quantified, and homogenized to form a sequencing library (SMRT Bell). The constructed library was first inspected, then the qualified library was sequenced using PacBio sequencing. PacBio Sequel offline data are in the Bam format. The circular consensus sequencing (CCS) file was exported through SMRT Link analysis software. Data from different samples were identified based on their barcode sequence and were converted into FASTQ format data.

The third-generation microbial diversity is based on the PacBio sequencing platform using the single-molecule real-time (SMRT) sequencing method to sequence the marker gene. This is followed by filtering, clustering, or denoising the sequence derived from CCS, and species annotation and abundance analysis, revealing the species composition of samples. Furthermore, alpha diversity (α) and beta diversity (β) analyses, correlation analysis, functional prediction, etc., can reveal the differences between samples.

The quality of the data is evaluated by processing parameters such as the number of samples and the length of the sequences in each stage. USEARCH (version 10.0) software was used to cluster the reads at a 97.0% similarity level to obtain the operational taxonomic units (OTUs). Classification information of each taxon can be obtained by using the naive Bayes classifier, combined with the alignment method, using SILVA as the reference database. Then, the community composition of each sample can be calculated at each level (phylum, class, order, family, genus, and species). The species abundance table at different classification levels can be generated using QIIME (version 2020.6) software. Last, the community structure map of the samples at each taxonomic level was drawn using R(4.1.0).

Taxonomic annotation of feature sequences was processed by Bayesian classifier and blast using SILVA(Release138, wwwarb-silva.de.) as the reference database. Statistics on composition in each sample were calculated at the level of phylum, class, order, family, genus, and species. QIIME was applied to obtain the abundance of each species in samples, and a distribution histogram at each taxonomic level was generated by a certain R package.

Alpha diversity metrics were evaluated by QIIME2 (version 2020.6), and Beta diversity analysis was processed by QIIME software to compare species diversity between different samples. There are four commonly used statistical algorithms to calculate the distance between samples in beta diversity analysis, that is binary Jaccard, https://en.wikipedia.org/wiki/Bray%E2%80%93Curtis_dissimilarity Bray–Curtis, weighted UniFrac (bacteria only), and https://en.wikipedia.org/wiki/UniFrac unweighted unifrac (bacteria only). These four algorithms can be classified into weighted (Bray–Curtis and weighted UniFrac) and non-weighted (Jaccard and unweighted UniFrac). The unweighted algorithm focuses on the existence of a species, while the weighted algorithm takes both existence and abundance into consideration.

### 16S functional genes prediction

#### Tax4fun function prediction

PICRUSt2 was applied to perform species annotation on sequences based on the reference phylogenetic tree. Potential functions and functional genes in samples were predicted based on the Integrated Microbial Genomes database, which further revealed differences in functions between samples or groups (Parks et al., 2014). The significant differences in function abundance between samples were evaluated by the G-test (the number of annotated functional genes >20) and Fisher (the number of annotated functional genes <20) using STAMP software (version 2.1.3) (Parks et al., 2014). The threshold for significant differences was set at a *p* < 0.05.

#### BugBase phenotype prediction

First, BugBase normalizes OTU based on the predicted 16S rRNA copy number. Microbial phenotype is predicted based on given pre-calculated files (Ward et al., 2017). For biological data of each sample, the relative abundance of the trait is estimated in the full range of coverage thresholds (0–1, increment: 0.01). Subsequently, for each trait, the coverage threshold with the highest variance in all samples is selected. With such a threshold, BugBase can generate a table with organism-level phenotype predictions, which contains a relative abundance of predicted phenotypes in each sample. Based on specified metadata, an automated hypothesis test is performed and can be visualized as taxa-contribution plots depicting the relative abundances of trait-possessing taxa. Outputs include summary files of non-parametric differentiation tests (Mann–Whitney U-test or Kruskal–Wallis test).

### Statistical analysis

Statistical analysis was performed using the R (4.1.0) Hmisc package (Frank, 2021). The correlation between serum parameters and genus and species level of gut microbes was assessed using Spearman's correlations. To verify the effect of serum parameters on microbiota, the correlation between serum parameters and clusters of orthologous group (COG) richness, gene richness, Shannon index, and β diversity in six adult Sunda pangolins were analyzed.

## Results

### Sequencing data

We generated 67,780 filtered and highly effective CCS 16S rRNA gene sequences (1,463 sequences). The number of sequences per sample ranged between 5,171 and 7,095 CCS (Supplementary Table 2). The rarefaction curves indicated that when the sequencing depth reached 6,000 sequences, the OTU number stopped increasing (Supplementary Figure 1A). These reads were assigned to 864 unique OTUs, with the number of OTUs per sample ranging from 52 to 132. For adult Chinese pangolins (group 1), 115 OTUs were classified into four and six phyla, 22 and 28 families, and 52 and 63 species, respectively (Supplementary Table 3). For adult Malayan pangolins (group 2), 617 OTUs were classified into an average of six phyla (ranging from 4 to 9), 37 families (ranging from 28 to 40), and 90 species (ranging from 72 to 130). For sub-adult Malayan pangolins (group 3), 209 OTUs were classified into four and 11 phyla, 29 and 69 families, and 77 and 133 species, respectively (Table 1).

**Figure 1:**
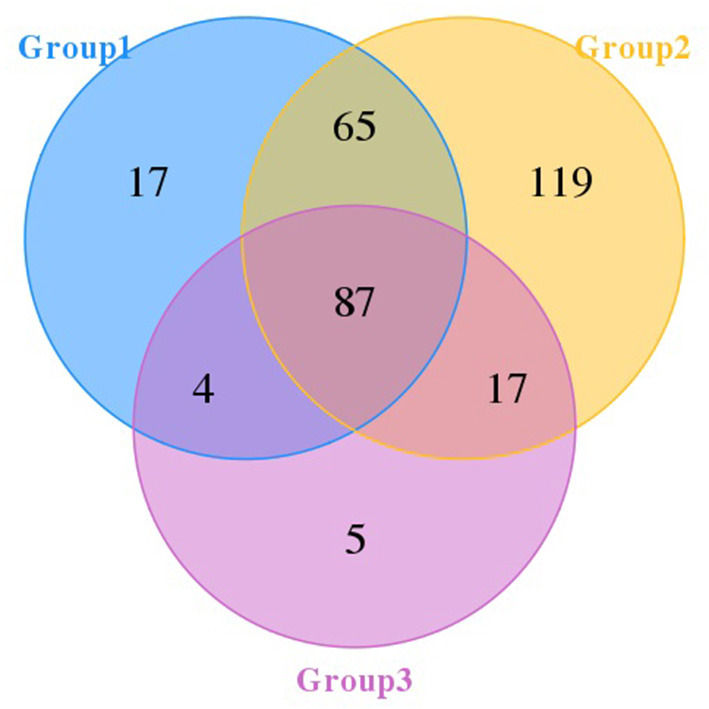
Venn diagram of pangolins. The number in each independent or overlapped area represents the number of unique or common features in each corresponding collection.

The Venn diagram (Figure 1) showed that there were 87 OTUs shared among the three groups. Groups 1, 2, and 3 had 17, 119, and 5 OTUs, respectively. Additionally, groups 1 and 2 shared 65 OTUs, and groups 2 and 3 shared 17 OTUs.

**Figure 2:**
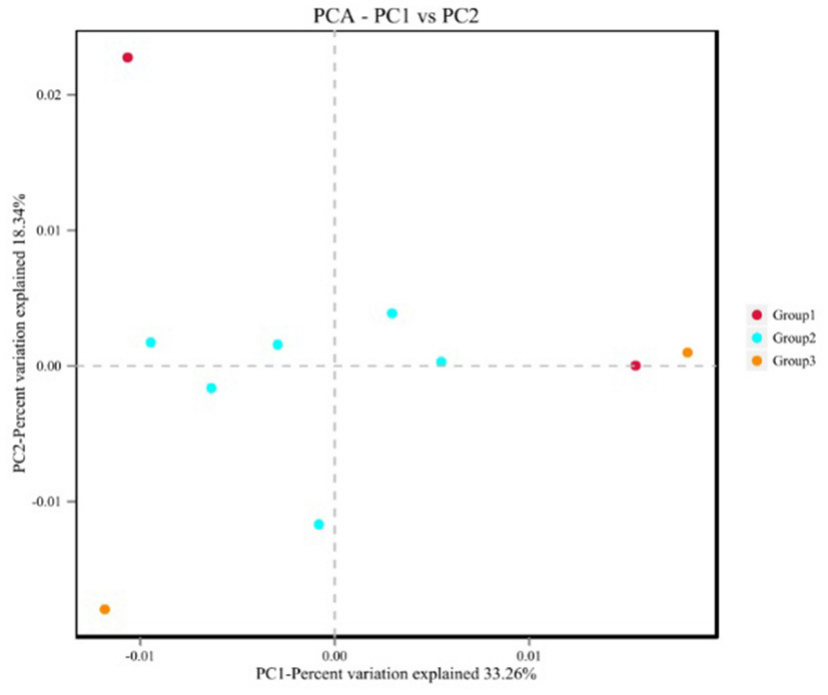
Principal component analysis graph.

The α diversity is shown in Supplementary Table 4, and the analysis of variance showed that there were no significant differences among the three groups. Principal component analysis (Figure 2) showed that group 2 showed obvious aggregation when separated from groups 1 and 3.

### Species and age cause different compositions of gut microorganisms

**Figure 3:**
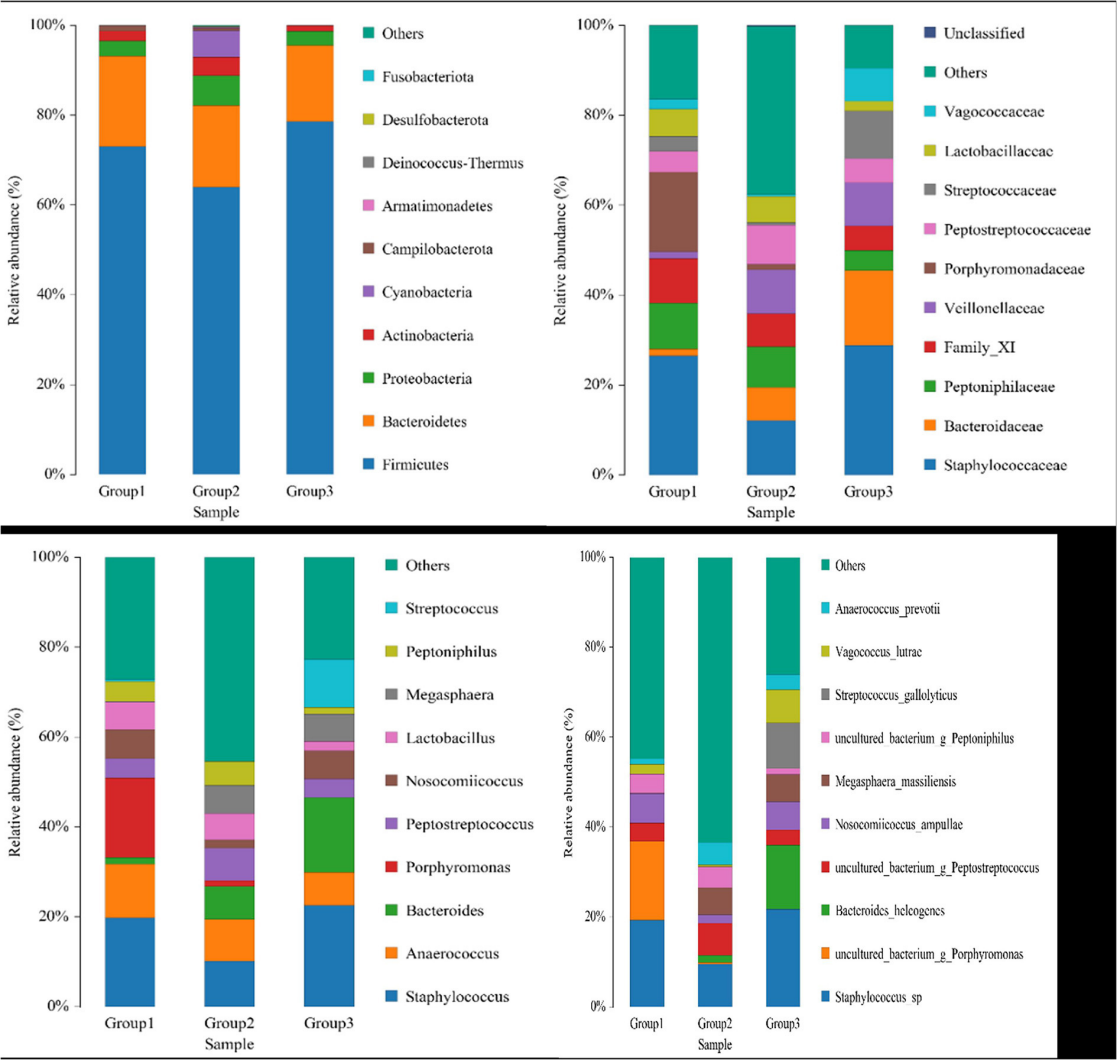
Species distribution in samples across the three groups.

The dominant phyla in the three groups were Firmicutes, Bacteroidetes, and Proteobacteria (Figure 3). The ratios of Firmicutes to Bacteroidetes in the three groups, groups 1, 2, and 3, were 3.64, 3.53, and 4.65, respectively. The 10 most abundant microbial families were *Staphylococcaceae, Bacteroidaceae, Peptoniphlaceae*, Family_ XI, *Veillonellaceae, Porphyromonadaceae, Peptostreptococcaceae, Streptococcaceae, Lactobacillaceae*, and *Vagococcaceae* (Figure 3). The most dominant family was *Staphylococcaceae*, but the proportion was higher in groups 1 and 3 but relatively lower in group 2. The top 10 genera abundance were *Staphylococcus, Anaerococcus, Bacteroides, Porphyromonas, Peptostreptococcus, Nosocomiicoccus, Lactobacillus, Megasphaera, Peptoniphilus*, and *Streptococcus* (Figure 3). The dominant genera in group 1 were *Staphylococcus, Porphyromonas*, and *Anaerococcus*; the dominant genera in group 2 were *Staphylococcus, Anaerococcus*, and *Bacteroides*; and the dominant genera in group 3 were *Staphylococcus, Bacteroides*, and *Streptococcus*.

The top 10 abundant species were *Staphylococcus* sp., *Porphyromonas* sp., *Bacteroides helcogenes, Peptostreptococcus* sp., *Nosocomiicoccus ampullae, Megasphaera massiliensis, Peptoniphilus* sp., *Streptococcus gallolyticus, Vagococcus lutrae*, and *Anaerococcus prevotii* (Figure 3). The dominant species in group 1 were *Staphylococcus* sp., *Porphyromonas* sp., and *Nosocomiicoccus ampullae*. The dominant species in group 2 were *Staphylococcus* sp., *Peptostreptococcus* sp., and *Megasphaera massiliensis*. The dominant species in group 3 were *Staphylococcus* sp., *Bacteroides helcogenes*, and *Streptococcus gallolyticus*. At the genus and species levels, the proportion of dominant species in group 2 was lower than that in groups 1 and 3, because the microbial species in group 2 were more abundant, and other species with lower proportions occupied the rest, which was more obvious at the species level. Eleven bacterial species that are related to immune cell development and function were identified in the animals included in the present study, including *Bacteroides coagulans, Bacteroides fragilis, Bacteroides* sp., *Bacteroides vulgatus, Bifidobacterium longum, Bifidobacterium pseudocatenulatum, Clostridium symbiosum, Parabacteroides distasonis, Parabacteroides merdae, Peptoniphilus harei*, and *Ruminococcus gnavus*. But these bacteria account for <5% of all microorganisms.

**Figure 4:**
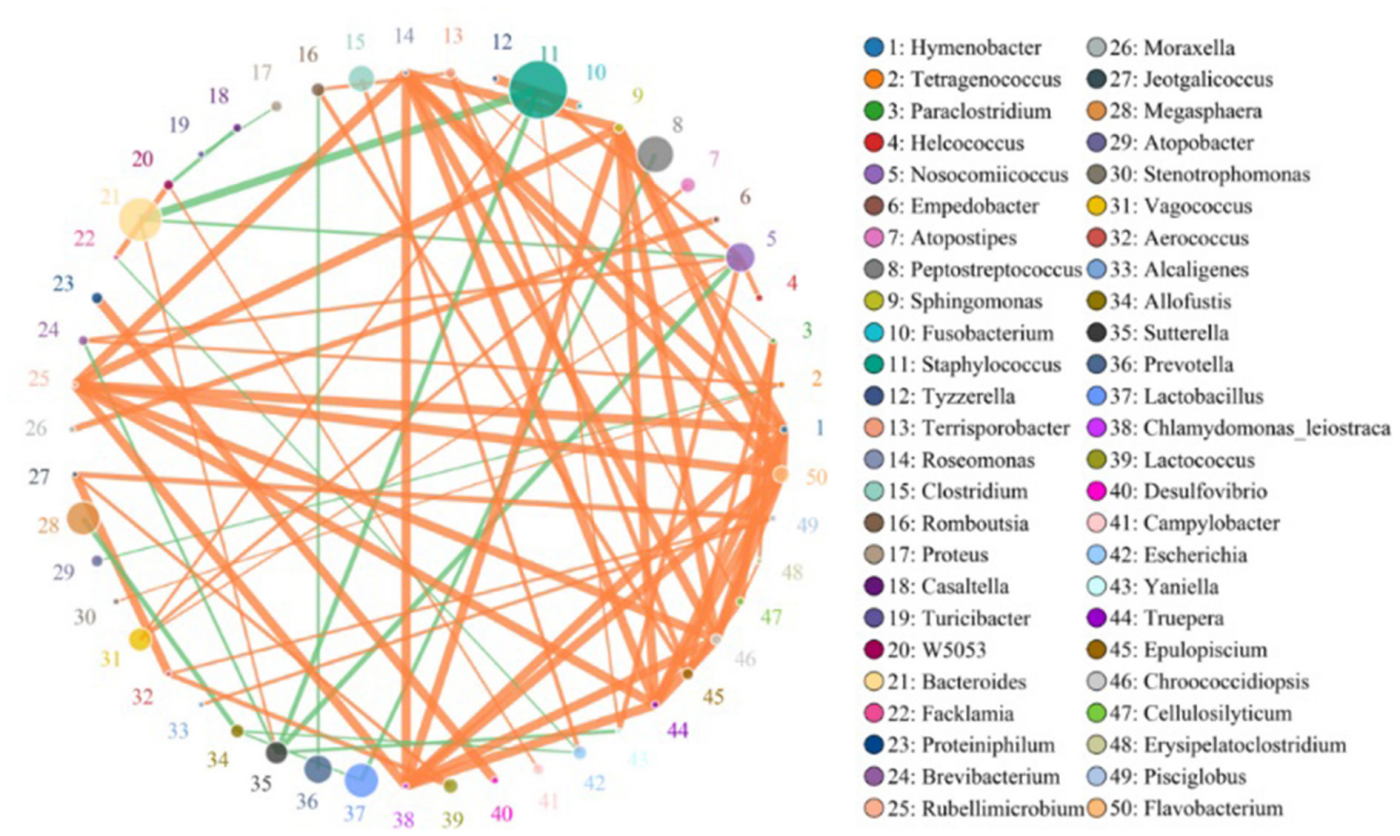
Species network at the genus level. Circles represent species; the size of the circle represents abundance; edges represent the correlation between the two species; thickness of the edge represents the strength of the correlation; and the color of the line represents correlation: orange represents positive correlation, while green represents negative correlation.

The species correlation network diagram, the top 50 genera with the highest correlation, is shown in Figure 4. *Staphylococcus, Peptostreptococcus, Lactobacillus, Bacteroides, Prevotella, Clostridium, Sutterella*, and *Megasphaera* were the most abundant genera and there were more positive and higher correlations between any two species.

### Functional diversity analysis

**Figure 5:**
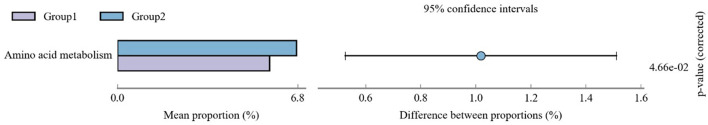
Differences between metabolic pathways.

Tax4fun prediction showed the most dominant metabolic pathways, including amino acid metabolism, xenobiotic biodegradation and metabolism, glycan biosynthesis and metabolism, replication and repair, translation, lipid metabolism, nucleotide metabolism, energy metabolism, cellular community—prokaryotes, metabolism of cofactors and vitamins, signal transduction, amino acid metabolism, membrane transport, carbohydrate metabolism, and global and overview maps. There were significant differences only in the amino acid metabolic pathways between groups 1 and 2 (Figure 5).

**Figure 6:**
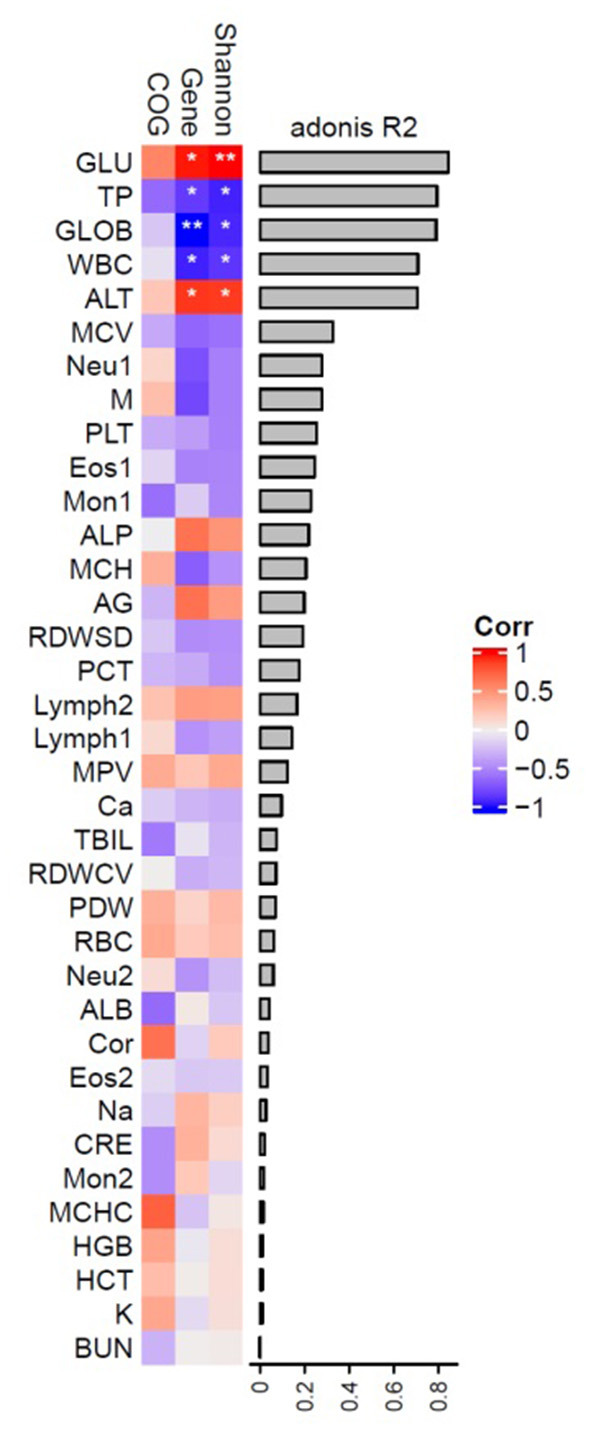
Busbage phenotypic prediction.

The prediction results of the BusBage phenotype showed that there were significant differences based on phenotypes among the three groups, mainly in phenotypes associated with aerobism, anaerobism, biofilm formation, genetic potential, and stress tolerance. Group 2 (adult Sunda pangolins) had a higher microbial abundance and proportion (Figure 6). The microbial species with different phenotypes unique to group 2 at the genus and species level are shown in Supplementary Table 5.

### Blood parameters and correlation analysis with microbiota

The assessment of pangolin blood parameters is shown in Table 2. The mean cortisol value was 643.47 ± 457.31. After grouping, the mean values of each group were significantly different. The cortisol level of adult Sunda pangolins in group 2 was the highest (849.65 ± 4), followed by that of sub-adult Sunda pangolins (562.2 ± 10). The cortisol level of adult Chinese pangolins was the lowest at 106.2 ± 15. The correlation between cortisol and blood parameters of six adult Sunda pangolins showed that cortisol was negatively associated with monocyte count (MON), monocyte percentage (MON %), albumin (ALB), and creatinine (CRE) significantly (Supplementary Table 6).

**Table 2:** Blood biochemical parameters of pangolin scales.

**Parameters**	**Mean ±SD**
WBC 10^9^/L	6.90 ± 1.92
Neu# 10^9^/L	1.71 ± 1.14
Eos# 10^9^/L	0.02 ± 0.01
lymph# 10^9^/L	4.40 ± 1.54
Mon# 10^9^/L	0.78 ± 0.94
Neu% %	25.29 ± 15.87
Eos% %	0.27 ± 0.18
Lymph% %	64.25 ± 16.23
Mon% %	10.19 ± 9.67
RBC 10^12^/L	7.10 ± 0.77
HGB g/L	155.30 ± 17.27
MCV fL	67.12 ± 3.84
MCH pg	21.91 ± 1.28
MCHC g/L	326.70 ± 7.36
RDW-CV %	13.12 ± 1.27
RDW-SD fL	34.20 ± 3.39
HCT %	47.51 ± 4.69
PLT 10^9^/L	97.30 ± 63.57
MPV fL	6.89 ± 1.57
PDW	16.24 ± 0.58
PCT %	0.06 ± 0.03
ALT	101.20 ± 35.48
TP	69.83 ± 7.05
ALB	30.09 ± 3.19
TBIL	1.02 ± 3.47
ALP	332.80 ± 360.51
GLU	2.87 ± 0.55
BUN	7.76 ± 1.55
CRE	53.40 ± 11.99
Na	158.12 ± 3.32
K	7.08 ± 1.15
GLOB	39.74 ± 4.50
A/G	0.76 ± 0.07
Ca	2.30 ± 0.31
Cor	643.47 ± 457.31
LgM	0.70 ± 0.26

A/G, albumin/globulin; ALB, albumin; ALP, alkaline phosphatase; ALT, alanine aminotransferase; BUN, blood urea nitrogen; Ca, calcium; Cor, cortisol; CRE, creatinine; CV, coefficient of variation; Eos, eosinophil; GLOB, globulin; GLU, glucose; HCT, hematocrit; HGB, hemoglobin; K, potassium; Lymph, lymphocyte; MCH, mean corpuscular hemoglobin; MCHC, mean corpuscular hemoglobin concentration; MCV, mean corpuscular volume; Mon, monocyte; MPV, mean platelet volume; Na, sodium; Neu, neutrophil; PCT, platelet hematocrit; PDW, platelet distribution width; PLT, platelet; RBC, red blood cells; RDW, red cell distribution width; SD, standard deviation; TBIL, total bilirubin; TP, total protein; WBC, white blood cells.

**Figure 7:**
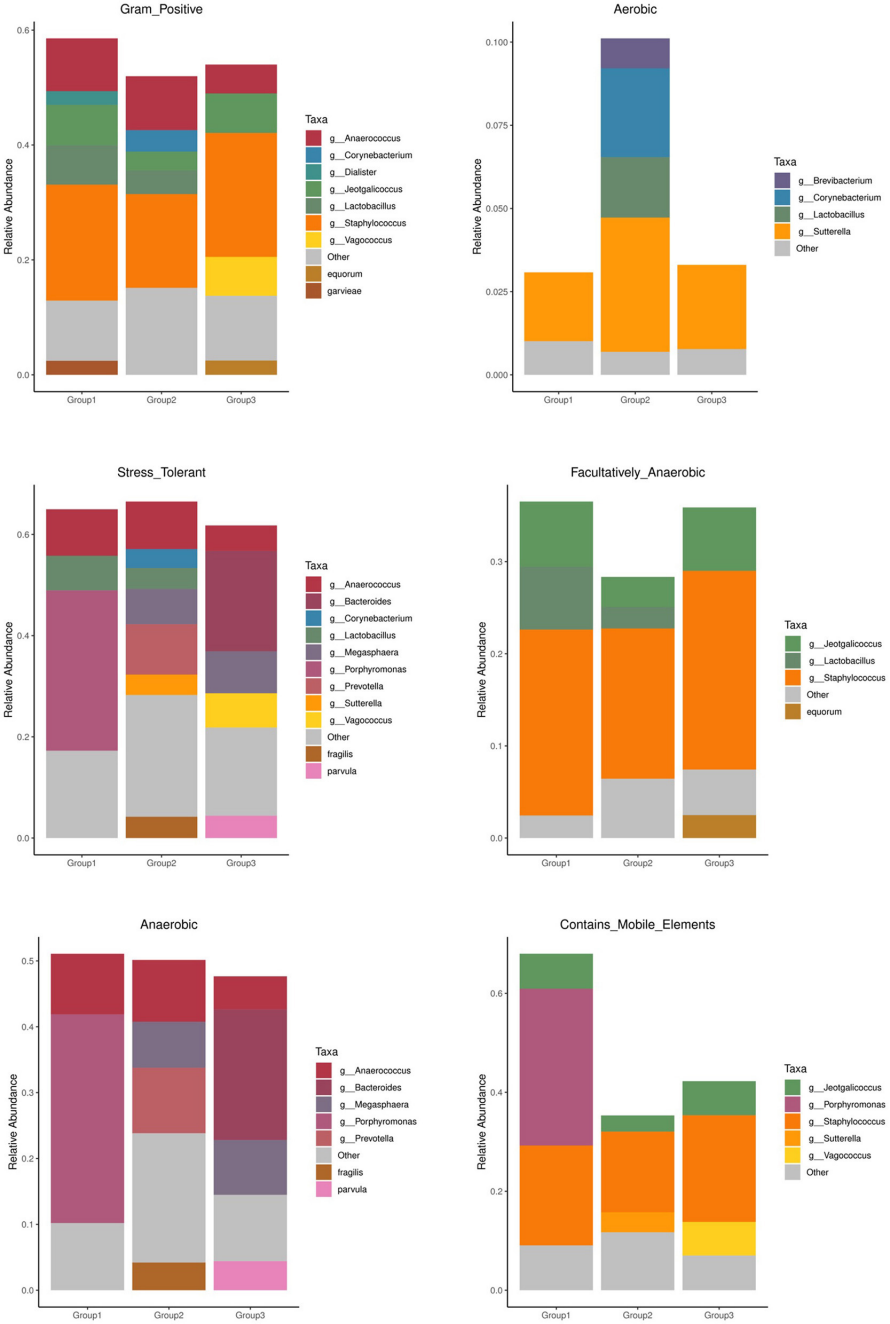
Intrinsic factors associated with inter-individual variation in the gut microbiome.

We correlated blood parameters with the interindividual of adult Sunda pangolins variation in microbial composition, diversity, the richness of genes, and COGs (Supplementary Figure 3B). As shown in Figure 7, the blood glucose (GLU), total protein (TP), globulin (GLOB), white blood cell (WBC) count, and alanine aminotransferase (ALT) explained β diversity of microbial community more than 0.8 and is related to the Shannon index and genetic diversity to varying degrees. A highly significant positive correlation exists between α diversity and gene diversity (*p* < 0.01, *p* < 0.05); TP and WBC are both highly negatively correlated with α diversity and gene diversity significantly; GLOB was significantly correlated with gene diversity and alpha diversity; ALT was significantly negatively correlated with α diversity and gene diversity.

## Discussion

The results showed that there was no significant difference in α diversity between adult Sunda pangolins and adult Chinese pangolins or between adult Sunda pangolins and sub-adult Sunda pangolins. However, there were significant differences in β diversity, indicating that there were differences in the intestinal microbial composition of different pangolins. The dominant genera in group 1 were *Staphylococcus, Porphyromonas*, and *Anaerococcus*; the dominant genera in group 2 were *Staphylococcus, Anaerococcus*, and *Bacteroides*; and the dominant genera in group 3 were *Staphylococcus, Bacteroides*, and *Streptococcus*. This was different from previous studies that reported *Lactobacillus, Escherichia*/*Shigella*, and *Clostridium sensu stricto* 1 as the dominant genera (Ma et al., 2018; Liu et al., 2021; Yan et al., 2021; Zhang et al., 2021). From now on, this is the first study to identify the dominant intestinal microbial species in pangolins using 16S full-length sequencing. Additionally, some bacterial species have been identified that could promote immune cell development (Li et al., 2021; Paik et al., 2022), of which, 11 bacterial species were identified in the pangolin gut; however, these bacteria account for <5% of all microorganism in every sample.

The BusBage phenotype prediction results showed significant differences among the three groups. Adult Sunda pangolin contained more abundant microbiota of several phenotypes, such as aerobism, anaerobism, biofilm formation, genetic potential, and stress tolerance (Figure 4), which was consistent with the result of previous studies. The differences in microorganisms between research subjects are not explained through microbial diversity, but mainly through functional diversity (Engel et al., 2012). Although for most animals, the species and age are the key factors affecting intestinal microorganisms (Li et al., 2018). The results of this study show that adult Sunda pangolins contain a more abundant microbiota to cope with environmental changes and stress. According to the record from IUCN, the Sunda pangolin is widely distributed in the wild, across the mainland and islands of Southeast Asia. In contrast, the Chinese pangolin is found in the Himalayan foothills of Nepal, southern Bhutan, and China, although China comprises the largest range state for this species (https://www.iucnredlist.org/). Therefore, the wider distribution in the history of Sunda pangolin may be the reason for the greater functional diversity of intestinal microorganisms.

Blood parameters were in accordance with previous research (Ahmad et al., 2018). The serum cortisol levels showed a significant difference in cortisol levels among the three groups. Some studies have shown that captive breeding causes an increase in pangolin cortisol levels (PCT: 72.04 ± 13, PCB: 158.01 ± 3) and changes the intestinal microbial diversity and structure (Yan et al., 2021). However, the results in this study are much higher than those of previous studies, which could be a result of long-term captive breeding. A large number of studies have shown that in different research subjects, the increase in cortisol has a significant correlation with some gut microbes. For example, *Bacteroidea* and γ-*Proteus, Bifidobacteria* and *Streptococcus, Staphylococcus*, and *Prevotella* (early infant) have a positive correlation with cortisol; whereas Firmicutes/*Bacteroides*, Actinomycetes, and *Lactobacillus* are negatively related to cortisol (Tillisch et al., 2013). The results of this study show that the dominant genera of the intestinal microorganisms in the three groups of pangolins are *Staphylococcus* and *Streptococcus*, which is not consistent with the results of previous studies while these species are positively correlated with cortisol levels. This indicates that increased serum cortisol changed the structure of intestinal microbial in pangolins caused by long-term stress.

The GLU, TP, GLOB, WBC, and ALT on the β diversity of pangolin gut microbiota are more than 0.8 and are related to the Shannon index and genetic diversity to varying degrees. As we know that GLU is an important component of the organism as the source of energy, TP has many functions such as maintaining normal colloid osmotic pressure and PH, transporting various metabolites, regulating the physiological function of transported substances, and relieving their toxicity, immune function and nutritional function; GLOB is a serum protein that exists in the body and has an immune function; WBC: when bacteria invade the human body, white blood cells can pass through the capillary wall through deformation, concentrate on the invasion site of bacteria, and surround and engulf the bacteria; ALT is an important indicator of liver function problems. These are important indicators of immunity. This may indicate that the diversity of intestinal microorganisms is closely related to immunity. The Spearman correlation analysis between cortisol level and MON (10^9^/L), MON%, ALB, and GRE showed that cortisol was negatively correlated with the parameters representing body immunity. This indicated that there is a correlation between cortisol and immunity and that an abnormal increase in cortisol may result in a decrease in pangolin immunity. Therefore, there may be an interaction between cortisol, immunity, and intestinal microorganisms. The study on piglets found that serum cortisol mediated the change between intestinal fecal rumen cocci and brain NAA (Mudd et al., 2017), which further confirmed that the change in dominant intestinal bacteria of pangolin was probably caused by the increase of cortisol, which also led to the reduction of pangolin immunity.

It is difficult to obtain samples from wild pangolins because intestinal microorganisms are affected by various factors, such as environment and diet. Therefore, although the sample size of this study is small, it can still be used as a basis for in-depth research. In conclusion, the results of this study show that captive adult Sunda pangolins have greater functional diversity of intestinal microorganisms and better adapt to environmental changes and stress. Therefore, increasing the levels of these microbes in Chinese pangolins in breeding programs can improve their adaptability to the environment, ultimately improving the health of breeding pangolins. Moreover, the high content of cortisol in pangolins, caused by captivity, may lead to insufficient immunity and alternation in the composition of the intestinal microbiota. Therefore, *Bifidobacteria* and bacterial species mentioned above are antagonistic to cortisol levels and can be increased in pangolins during breeding to reduce the stress response. In the present study, sub-adult Sunda pangolins had the lowest OTU specificity compared to that of the other groups. This perhaps attributes to age; however, because this group of pangolins was born in captivity, we cannot rule out whether the low OTU specificity was caused by a lack of contact with the natural habitat. This finding requires further in-depth study.

## Data availability statement

The datasets presented in this study can be found in on line repositories. The names of the repository/repositories and accession number(s) can be found at: https://www.ncbi.nlm.nih.gov/, PRJNA842545.

## Ethics statement

The procedures for care and use of animals were approved by the Ethics Committee of the Institute of Zoology, Guangdong Academy of Sciences (Guangzhou, China) and all applicable institutional and governmental regulations concerning the ethical use of animals were followed.

## Author contributions

WJ accomplish sampling, experiment, data analysis, and paper writing. LLiu and ZZ accomplish sampling. LLi accomplish part of analysis and sampling. JC accomplish experimental design. All authors contributed to the article and approved the submitted version.

## Funding

This study was supported by the Biodiversity Maintenance Mechanism and Regional Biosafety Innovation Research in Lingnan (2022GDASZH-2022010106).

## Conflict of interest

The authors declare that the research was conducted in the absence of any commercial or financial relationships that could be construed as a potential conflict of interest.

## Publisher's note

All claims expressed in this article are solely those of the authors and do not necessarily represent those of their affiliated organizations, or those of the publisher, the editors and the reviewers. Any product that may be evaluated in this article, or claim that may be made by its manufacturer, is not guaranteed or endorsed by the publisher.
