# Supplementary material for: Differences in gut microbes in captive pangolins and the effects of captive breeding
Source: Front Microbiol. 2022 Dec 6;13:1053925. doi: 10.3389/fmicb.2022.1053925 (PMC9763570; doi:10.3389/fmicb.2022.1053925)
Supplement: Supplementary file 1 [file Data_Sheet_1.docx]

Table S1 Sample details and grouping

| Grouping | Sample | Weight(kg) | Sex | Species |
| --- | --- | --- | --- | --- |
| Group1 | SZS1 | 6.34 | male | *Manis pentadactyla* |
|  | SZS2 | 5.98 | male | *Manis pentadactyla* |
| Group2 | SZS3 | 6.59 | male | *Manis javanica* |
|  | SZD1 | 5.34 | female | *Manis javanica* |
|  | SZD3 | 6.2 | male | *Manis javanica* |
|  | SZ03 | 5.16 | male | *Manis javanica* |
|  | SZ11 | 3.99 | female | *Manis javanica* |
|  | SZ15 | 5.39 | male | *Manis javanica* |
| Group3 | SZXWZ | 2.56 | male | Sub*-Manis javanica* |
|  | SZXTY | 1.52 | female | Sub*-Manis javanica* |

Table S2 Summary on raw data processing

|  | Sample ID | Raw CCS | Clean CCS | Effective CCS | AvgLen(bp) | Effective(%) |
| --- | --- | --- | --- | --- | --- | --- |
| Group1 | SZS1 | 7,510 | 7,077 | 7,073 | 1,458 | 94.18 |
|  | SZS2 | 7,521 | 7,026 | 7,011 | 1,473 | 93.22 |
| Group2 | SZS3 | 7,478 | 6,983 | 6,955 | 1,476 | 93.01 |
|  | SZD1 | 7,502 | 6,945 | 6,916 | 1,465 | 92.19 |
|  | SZD3 | 5,551 | 5,195 | 5,171 | 1,454 | 93.15 |
|  | SZ03 | 7,593 | 7,101 | 7,095 | 1,462 | 93.44 |
|  | SZ11 | 7,514 | 6,951 | 6,882 | 1,448 | 91.59 |
|  | SZ15 | 7,481 | 6,864 | 6,800 | 1,452 | 90.9 |
| Group3 | SZXWZ | 7,486 | 6,930 | 6,875 | 1,463 | 91.84 |
|  | SZXTY | 7,497 | 7,013 | 7,002 | 1,476 | 93.4 |

Table S3 Statistics of species annotation

| Sample | Phylum | Class | Order | Family | Genus | Species |
| --- | --- | --- | --- | --- | --- | --- |
| SZS1 | 6 | 10 | 19 | 28 | 42 | 63 |
| SZS2 | 4 | 7 | 14 | 22 | 33 | 52 |
| SZS3 | 6 | 10 | 16 | 28 | 49 | 72 |
| SZ03 | 4 | 10 | 22 | 41 | 65 | 113 |
| SZ11 | 5 | 9 | 20 | 33 | 49 | 75 |
| SZ15 | 9 | 15 | 29 | 45 | 59 | 76 |
| SZD1 | 5 | 10 | 19 | 35 | 51 | 74 |
| SZD3 | 5 | 9 | 22 | 40 | 75 | 130 |
| SZSWZ | 4 | 8 | 20 | 29 | 51 | 77 |
| SZXTY | 11 | 20 | 44 | 69 | 101 | 132 |
| Total | 14 | 24 | 52 | 93 | 173 | 274 |

Table S4 Summary of alpha diversity

|  | ID | Feature | ACE | Chao1 | Simpson | Shannon |
| --- | --- | --- | --- | --- | --- | --- |
| Group1 | SZS1 | 68 | 71.42 | 75.5 | 0.95 | 4.84 |
|  | SZS2 | 56 | 61.38 | 59.75 | 0.85 | 3.7 |
| Group2 | SZS3 | 82 | 96.65 | 92.46 | 0.81 | 3.32 |
|  | SZ03 | 125 | 147.46 | 148.4 | 0.88 | 4.29 |
|  | SZ11 | 83 | 94.25 | 91.67 | 0.9 | 4.38 |
|  | SZ15 | 82 | 82.2 | 82 | 0.95 | 5.05 |
|  | SZD1 | 86 | 94.94 | 103.5 | 0.85 | 3.83 |
|  | SZD3 | 146 | 172.33 | 177 | 0.84 | 4.11 |
| Group3 | SZSWZ | 86 | 108.1 | 100.62 | 0.9 | 4.18 |
|  | SZXTY | 142 | 159.79 | 165.4 | 0.85 | 4.03 |

Table S5 Bacteries particular to adult *Manis javanica*by Busbage prediction

| Function | Genus: |
| --- | --- |
| Aerobic： | Brevibacterium、Corynebacterium、Lactobacillus |
| Anaerobic | Bacteroides、Megasphaera |
| Forms Biofilms | Bifidobacterium、Brevibacterium、Proteus |
| Potential pothgenic | Bacteroides、Megasphaera、Prevotella、Sutterella |
| Stress telorence | Bacteroides、Corynebacterium、Megasphaera、Prevotella、Sutterella |
| Function | Species |
| Aerobic | *g__Brevibacterium 、g__Corynebacterium 、g__Lactobacillus* |
| Anaerobic | *g__Megasphaera、g__Prevotella、fragilis* |
| Forms Biofilms | *g__Bifidobacterium 、g__Brevibacterium、 g__Proteus、morganii* |
| Gram negative | g__Megasphaera、g__Prevotella、g__Sutterella、fragilis |
| Potential pothgenic | g__Megasphaera、g__Prevotella、 g__Sutterella、fragilis |
| Stress telorence | g__Corynebacterium、g__Lactobacillus、g__Megasphaera、g__Prevotella、g__Sutterella、ragilis |

Table S6 The corticol and blood parameters corellation

|  | Mon 10^9^/L | Mon % | ALB | CRE |
| --- | --- | --- | --- | --- |
| r | -0.79 | -0.75 | -0.68 | -0.72 |
| p | 0.01 | 0.01 | 0.03 | 0.02 |


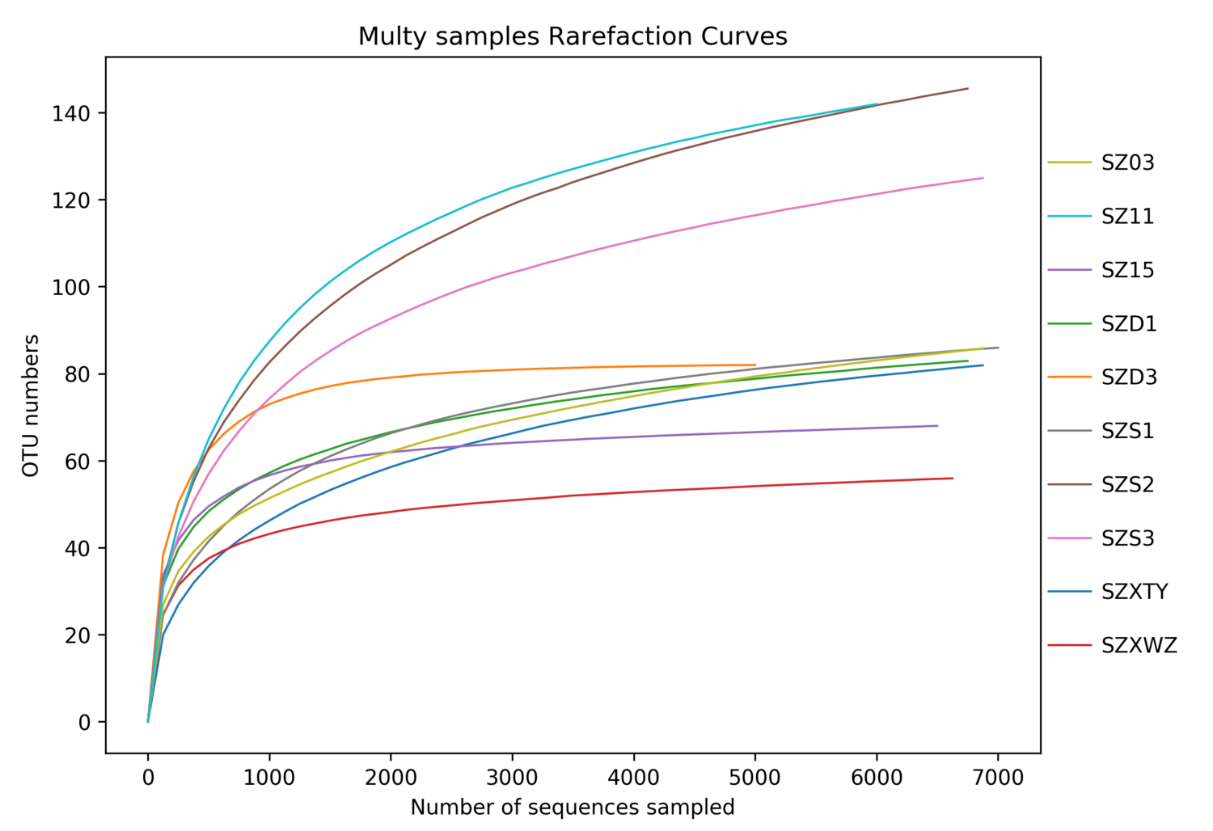

Figure S1 Multy samples Rarefaction Curves
